# Efficacy of a novel chewable tablet containing sarolaner, moxidectin and pyrantel (Simparica Trio™) against four common tick species infesting dogs in Europe

**DOI:** 10.1186/s13071-020-3949-y

**Published:** 2020-03-01

**Authors:** Csilla Becskei, Julian Liebenberg, Mirjan Thys, Sean P. Mahabir

**Affiliations:** 1 Zoetis, Veterinary Medicine Research and Development, Mercuriusstraat 20, 1930 Zaventem, Belgium; 2 Clinvet International (pty) Ltd, Uitsigweg, Bainsvlei 9338 Bloemfontein, Republic of South Africa; 3 Zoetis, Veterinary Medicine Research and Development, 333 Portage St., Kalamazoo, MI 49007 USA

**Keywords:** *Dermacentor reticulatus*, *Ixodes hexagonus*, *Ixodes ricinus*, Moxidectin, *Rhipicephalus sanguineus*, Sarolaner, Pyrantel

## Abstract

**Background:**

Tick infestations can cause direct deleterious effects to dogs as a result of tick blood-feeding, and indirectly ticks can transmit disease agents that can be detrimental to the health of both dogs and humans. Six laboratory studies were conducted to support dosage selection and efficacy confirmation of a novel combination of sarolaner, moxidectin and pyrantel against four tick species that commonly infest dogs in Europe.

**Methods:**

Two studies were conducted against *Dermacentor reticulatus* (one of which was a dose determination study), two against *Ixodes ricinus*, and one each against *Ixodes hexagonus* and *Rhipicephalus sanguineus* (*sensu lato*). In each study, eight purpose-bred Beagle or mix-breed dogs were randomly allocated to each treatment group and infested with 50 unfed adult ticks on Days-2, 5, 12, 19, 26 and 33. On Day 0 dogs were treated orally with placebo or the combination product. In the dose determination study, dogs received sarolaner at point dosages of 0.6 mg/kg, 1.2 mg/kg or 2.4 mg/kg in combination with moxidectin and pyrantel, and in all other studies dogs received Simparica Trio™ to provide minimum dosages of 1.2 mg/kg sarolaner, 24 µg/kg moxidectin and 5 mg/kg pyrantel (as pamoate salt). Efficacy was assessed based on live tick counts conducted 48 hours after treatment and each weekly infestation.

**Results:**

There were no treatment-related adverse events in any study. In the dose determination study, 1.2 mg/kg sarolaner was the lowest dosage evaluated that provided > 90% efficacy for at least 28 days and therefore was selected as the dosage to provide tick control for at least one month following a single oral treatment. In the dose confirmation studies, a single oral dose of Simparica Trio™ provided ≥ 99.2% efficacy against existing infestations of all tick species, and against re-infestations efficacy was ≥ 97.2% against *D. reticulatus* for 28 days and against all other species for 35 days.

**Conclusions:**

These studies support the sarolaner dose selected and confirm the efficacy of a single oral dose of Simparica Trio™ against existing infestations and re-infestations of the common tick species infesting dogs in Europe for at least one month.

## Background

Ticks are present throughout Europe with *Ixodes ricinus*, *Ixodes hexagonus*, *Dermacentor reticulatus* and *Rhipicephalus sanguineus* most often found infecting dogs [1]. Infestations cause both direct and indirect adverse clinical consequences to the dog; direct effects include nuisance, alopecia, skin irritation and in heavy infestations even anemia [2], and indirect effects are caused by the transmission of disease agents that can lead to serious and even life-threatening illness. *Ehrlichia canis* (causing canine ehrlichiosis), *Borrelia burgdorferi* (*sensu lato*) (causing Lyme borreliosis), *Anaplasma phagocytophilum* (causing granulocytic anaplasmosis), *Babesia canis* (causing babesiosis), *Hepatozoon canis* (causing hepatozoonosis) and *Rickettsia conorii* (causing Mediterranean spotted fever) are all transmitted to dogs by *Dermacentor*, *Ixodes* and *Rhipicephalus* ticks [3].

Due to the potentially severe consequences of tick infestation, any visible ticks should be removed from the dog as soon as possible after discovery, and treatment with acaricidal products is recommended for dogs found to be infested with ticks and/or living in or travelling to tick/tick-borne disease endemic regions [1, 4].

Dogs are however not only exposed to ticks but also often to infection by other ectoparasites, gastrointestinal and vascular nematodes. Both heartworm (*Dirofilaria immitis*) and lungworm (*Angiostrongylus vasorum*) may cause severe illness and potentially death in dogs, while gastrointestinal roundworms (*Toxocara canis*, *Toxascaris leonina*) and hookworms (*Ancylostoma caninum*, *Uncinaria stenocephala*) may not only cause clinical signs in the dogs but may also pose a zoonotic threat to humans as well [5]. Dogs therefore often require concurrent curative and/or preventive treatment against these parasites [1, 5]. A combination product containing not only sarolaner, a novel ectoparasiticide, but also moxidectin and pyrantel will provide comprehensive coverage against most of the parasites that commonly infect dogs and may pose health risks to humans.

The studies presented here assessed the minimum dose of sarolaner required in combination with moxidectin and pyrantel in a new oral chewable tablet (Simparica Trio™, Zoetis, Parsippany, NJ, USA) to provide efficacy for at least one month against the least susceptible European tick species, *D. reticulatus*, in dogs [6, 7]. The efficacy of the selected dose was further investigated against three additional tick species commonly infesting dogs in Europe.

## Methods

Six placebo controlled, masked, randomized laboratory studies were conducted to evaluate efficacy of the combination product against four tick species commonly infesting dogs in Europe: *D. reticulatus* (ornate dog tick; Studies 1 and 2); *I. hexagonus* (hedgehog tick; Study 3); *I. ricinus* (castor bean tick or sheep tick; Studies 4 and 5); and *R. sanguineus* (*s.l.*) (brown dog tick; Study 6). In addition to efficacy confirmation, Study 1 also served as the dose determination study for sarolaner against ticks. It has been reported previously that of these four tick species, *D. reticulatus* is the least susceptible to sarolaner, i.e. it is considered the dose limiting tick species for this active ingredient [6]. Therefore, the dose determination study was conducted using this species. All studies were conducted in accordance with the World Association for the Advancement of Veterinary Parasitology (WAAVP) and the European Medicine Agency guidelines for evaluating the efficacy of parasiticides for the treatment, prevention and control of flea and tick infestation on dogs and cats [8, 9], and complied with Good Clinical Practices [10]. Personnel involved in making assessments of efficacy or safety were masked to treatment assignments.

### Animals

Eight dogs were included in each treatment group. In Studies 1 and 5, short-haired, purpose bred mixed breed dogs were used; 15 males and 17 females in Study 1 and 9 males and 7 females in Study 5. In Studies 2, 3 and 4, purpose-bred Beagle dogs were used, 8 males and 8 females in each study. Animals ranged in age from 9 months to 7 years and weighed between 10.5 and 26.5 kg. Females were confirmed not to be pregnant or lactating. Dogs were individually identified by microchip and acclimated at the study facility for a minimum of 7 days prior to treatment. All dogs were assessed as being in good health at the time of enrollment based on physical examination by a suitably trained veterinarian. Dogs were housed in individual indoor pens such that no physical contact was possible between them. Dogs were fed an appropriate commercial diet and water was provided *ad libitum*. All dogs underwent an adequate wash-out period to ensure that no residual tick efficacy remained from any previously administered treatments. General health observations were performed daily from the start of the acclimation period until the end of the study.

### Design

The studies were designed to evaluate immediate efficacy against an infestation of ticks present on the dog at the time of treatment, and for persistent efficacy against tick re-infestations occurring weekly for 5 weeks after treatment. Therefore, dogs were infested with ticks 2 days before study treatment and at weekly intervals after treatment for 5 weeks. Efficacy was assessed based on live tick counts conducted 48 hours after treatment and 48 h after each subsequent tick infestation.

Day 0 for each study was defined as the day treatment was administered. On Day-7 or -8 each dog was examined to ensure it was free of ticks then infested with the respective tick species investigated in the individual study, in order to evaluate its suitability as a host for the tick species under evaluation. The live attached ticks present on each dog were counted and removed at 48 (± 2) hours after infestation. Dogs with the highest host-suitability tick counts were selected from the pool of available dogs and ranked by decreasing tick counts into blocks, and randomly allocated within block to treatment group. Blocks were randomly assigned to adjacent pens. Dogs were moved into their allocated pens on or before Day-2.

On Day 0, dogs were treated with placebo (Simparica Trio™ formulation without active ingredients) or the combination product (Simparica Trio™). Simparica Trio™ was provided in four different tablet strengths for each study. Each tablet of the different tablet strength contained the following quantities of sarolaner, moxidectin and pyrantel (as pamoate salt), respectively: 3/0.06/12.5 mg, 6/0.12/25 mg, 12/0.24/50 mg or 24/0.48/100 mg. Dose calculation of the three active ingredients was based on the body weight recorded on Day-2. In the *D. reticulatus* dose determination study (Study 1), efficacy of three different dosages of sarolaner (0.6 mg/kg, 1.2 mg/kg, and 2.4 mg/kg) in combination with moxidectin and pyrantel were evaluated. Entire Simparica Trio™ tablets containing uniform amounts of sarolaner were shaved and/or sanded in order to deliver the exact target sarolaner dosages. In all other studies dogs allocated to treatment with the combination product were dosed with single tablets or a combination of tablets of different strengths to receive as close as possible the minimum dosages of 1.2 mg/kg sarolaner + 24 µg/kg moxidectin + 5 mg/kg pyrantel (as pamoate salt) without under-dosing.

In all studies food was withheld overnight prior to treatment administration and was not offered again until approximately 4 h after treatment administration. Treatments were administered by hand pilling to ensure accurate dosing. Each dog was observed for several minutes after dosing for evidence that the dose was swallowed and for up to 2 h for evidence of vomiting. Dogs were observed for clinical signs 1, 3, 6 and 24 h after dosing.

### Tick infestations and tick counts

For efficacy evaluation, dogs were first infested with ticks on Day-2. Following treatment on Day 0, each dog was examined and combed to remove and count ticks 48 h later, on Day 2. Dogs were subsequently infested with ticks on Days 5, 12, 19, 26 and 33, with tick counts and removal conducted 48 h later, on Days 7, 14, 21, 28 and 35.

Ticks were obtained from laboratory-maintained colonies that were originally established using ticks collected from the field and subsequently enhanced by routine introduction of wild-caught field ticks.

For each tick infestation, a pre-counted aliquot of 50 ticks were placed on the hair coat and allowed to disperse on the dog. *Dermacentor reticulatus* and *R. sanguineus* were applied in an approximate 1:1 sex ratio, and *I. hexagonus* and *I. ricinus* in an approximate 3:2 female to male sex ratio [9]. Dogs were sedated for each infestation for approximately one hour by intramuscularly administered medetomidine hydrochloride according to the approved label dosage to enhance tick attachment.

Tick counts were performed by personnel trained in the standard procedures used at the test facility. Dogs were examined and combed to count ticks in a pre-determined random order. The dog’s entire body was first thoroughly visually examined, and ticks counted and removed. Counting began at the head and proceeded to cover all areas of the animal that could be examined with the dog in standing position, then the dog was gently turned on its back and the remaining areas were examined. After the manual inspection, an extra-fine-tooth comb was used to comb the dog to remove any remaining ticks. Each dog was examined for at least 10 min, if any ticks were encountered in the last minute, combing was continued in 1-min increments until no ticks were encountered. Personnel changed protective clothing and the table surface was cleaned between each dog to avoid any possible cross-contamination. The ticks were examined to assess viability and the numbers of live and dead ticks was quantified. Ticks were considered live if they showed any movement.

### Data analysis

The experimental unit was the individual dog and the primary endpoint was the live (free + attached) tick counts. Percent efficacy based on arithmetic live mean counts relative to placebo was calculated as follows:

$$\% {\text{Reduction = 100 }} \times \frac{{{\text{Mean count (placebo) }}{-} {\text{Mean count (treated)}}}}{\text{Mean count (placebo)}}$$

Tick counts were natural log transformed [log_e_ (count + 1)] prior to analysis. Transformed counts were analyzed using a mixed linear model for repeated measures using the PROC MIXED procedure (SAS 9.4, Cary NC). The model included the fixed effects of treatment, time-point, and treatment by time-point interaction. The random effects were block, the interaction between block and treatment (animal term), and error for all studies except Study 1 for which the random effects were room, block within room, and the interaction between block and treatment within room (animal term), and error. Testing was two-sided at the α = 0.05 significance level.

## Results

No abnormal health events related to treatment with the combination product were observed in any study.

### *Dermacentor reticulatus*

Placebo-treated dogs maintained adequate tick infestations throughout both studies with mean tick recoveries ranging from 37–73% of the applied infestation (Tables 1 and 2). In the dose determination study, all three dosages of sarolaner in the combination product provided ≥ 98.9% efficacy against an existing tick infestation. Against subsequent re-infestations, the 0.6 mg sarolaner dosage in the combination product provided efficacy of ≥ 95.6% on Days 7 and 14, but ≤ 87.4% from Days 21 to 35. In contrast, both the 1.2 and 2.4 mg/kg dosages provided ≥ 95.0% efficacy from Days 7 to Day 35 (Table 1). Mean tick counts for combination product-treated dogs at all 3 dosages of sarolaner were lower than those for placebo at all time-points (5.48 ≤ *t*_*df*_ ≤ 19.04, 29.3 ≤ *df* ≤ 44.7, *P* < 0.0001). There was no significant difference between the mean tick counts for the 3 sarolaner dosages through Day 14 (0.00 ≤ *t*_*df*_ ≤ 1.85, 28.8 ≤ *df* ≤ 61.4, *P* ≥ 0.0742), but these were significantly lower for both the 1.2 and 2.4 mg/kg sarolaner dosages in the combination product than the mean tick counts for the 0.6 mg/kg dosage on Days 21 through 35 (2.61 ≤ *t*_*df*_ ≤ 4.47, 28.8 ≤ *df* ≤ 37.4, *P* ≤ 0.0144). Mean tick counts for the 2.4 mg/kg dosage were significantly lower than those for the 1.2 mg/kg dosage on Day 35 (*t*_(61.4)_ = 2.74, *P* = 0.0080). These results indicated that 1.2 mg/kg was the minimum required dosage of sarolaner in the combination product to provide efficacy against the European dose limiting tick for at least one month following a single oral administration.

**Table 1:** Arithmetic mean live *Dermacentor reticulatus* counts for dogs dosed once orally with sarolaner + moxidectin + pyrantel pamoate and efficacy relative to placebo (Study 1)

Count day	Placebo	Sarolaner (mg/kg) + moxidectin (µg/kg) + pyrantel (mg/kg)					
	Mean	0.6 + 12 + 2.5		1.2 + 24 + 5		2.4 + 48 + 10	
		Mean	% Efficacy	Mean	% Efficacy	Mean	% Efficacy
2	34.1^a^	0.1^b^	99.6	0.2^b^	98.9	0^b^	100
7	28.6^a^	0.6^b^	95.6	0.1^b^	99.6	0.1^b^	99.6
14	25.8^a^	0.7^b^	96.6	0.2^b^	98.1	0^b^	100
21	23.8^a^	1.7^b^	87.4	0.2^c^	98.9	0.3^c^	97.9
28	23.8^a^	1.8^b^	85.8	0.2^c^	98.9	0^c^	100
35	25.3^a^	3.3^b^	79.7	0.9^c^	95.0	0.2^d^	99.0

*Note*: Dogs infested with ticks from colony originated from Ireland and subsequently enriched with ticks from the Netherlands, the last time 4 years before study conduct. Mean live tick counts with the same superscript within rows are not significantly different (*P* > 0.05)

**Table 2:** Arithmetic mean live *Dermacentor reticulatus* counts for dogs dosed once orally with sarolaner + moxidectin + pyrantel and efficacy relative to placebo (Study 2)

Count day	Placebo		1.2 mg/kg sarolaner + 24 µg moxidectin + 5 mg/kg pyrantel			Test statistic (*vs* placebo)
	Mean	Range	Mean^a^	Range	% Efficacy	
2	29.8	20–37	0.3	0–1	99.2	*t*_(14)_ = 23.43 *P* < 0.0001
7	36.5	30–45	0	0	100	*t*_(14)_ = 79.48 *P* < 0.0001
14	30.3	15–37	0.1	0–1	99.6	*t*_(14)_ = 24.22 *P* < 0.0001
21	33.6	20–47	0.1	0–1	99.6	*t*_(14)_ = 25.55 *P* < 0.0001
28	35.8	22–45	1.0	0–5	97.2	*t*_(14)_ = 11.42 *P* < 0.0001
35	24.7	18–36	3.9	0–15	84.3	*t*_(14)_ = 12.22 *P* < 0.0001

*Note*: Dogs infested with ticks from colony originated from the UK and subsequently enriched with ticks from Slovakia, Germany and the Netherlands, the last time 2 years before study conduct

^a^Mean live tick count significantly lower than placebo (11.42 ≤*t*_(14)_ ≤ 79.48, *P* < 0.0001) at all time points

In the dose confirmation study, efficacy against an existing infestation was 99.2%, and efficacy against subsequent weekly re-infestations was ≥ 97.2% through Day 28 and 84.3% on Day 35 (Table 2). Mean tick counts were significantly lower than placebo on all post-treatment counts (11.42 ≤ *t*_(14)_ ≤ 79.48, *P* < 0.0001).

### *Ixodes hexagonus*

Placebo-treated dogs maintained adequate tick infestations throughout the study with mean tick recoveries ranging from 32–39% of the applied infestation (Table 3). Efficacy against an existing infestation was 100% and efficacy against subsequent weekly re-infestations was ≥ 98.6% through Day 35. Mean tick counts were significantly lower than placebo on all post-treatment counts (17.16 ≤ *t*_*df*_ ≤ 63.54, 13.3 ≤ *df* ≤ 14.9, *P* < 0.0001).

**Table 3:** Arithmetic mean live *Ixodes hexagonus* counts for dogs dosed once orally with sarolaner + moxidectin + pyrantel and efficacy relative to placebo (Study 3)

Count day	Placebo		1.2 mg/kg sarolaner + 24 µg moxidectin + 5 mg/kg pyrantel			Test statistic (*vs* placebo)
	Mean	Range	Mean^a^	Range	% Efficacy	
2	16.5	10–27	0	0	100	*t*_(14.6)_ = 29.09 *P* < 0.0001
7	17.5	8–24	0	0	100	*t*_(14.7)_ = 21.55 *P* < 0.0001
14	17.4	9–25	0	0	100	*t*_(14.9)_ = 24.93 *P* < 0.0001
21	16.6	13–21	0	0	100	*t*_(13.3)_ = 50.30 *P* < 0.0001
28	19.5	15–23	0	0	100	*t*_(13.9)_ = 63.54 *P* < 0.0001
35	17.6	12–26	0.3	0–2	98.6	*t*_(13.9)_ = 17.16 *P* < 0.0001

*Note*: Dogs infested with ticks from colony originated with ticks from Belgium the same year as study conduct

^a^Mean live tick count significantly lower than placebo (17.16 ≤ *t*_*df*_ ≤ 63.54, 13.3 ≤ *df* ≤ 14.9, *P* < 0.0001) at all time-points

### *Ixodes ricinus*

Placebo-treated dogs maintained adequate tick infestations throughout the studies with mean tick recoveries ranging from 28–36% of the applied infestation (Table 4). In both studies, efficacy against an existing infestation was 100% and efficacy against subsequent weekly re-infestations was ≥ 97.2% through Day 35. Mean tick counts were significantly lower than placebo on all post-treatment counts (13.25 ≤ *t*_*df*_ ≤ 63.54, 9.4 ≤ *df* ≤ 15.3, *P* < 0.0001).

**Table 4:** Arithmetic mean live *Ixodes ricinus* counts for dogs dosed once orally with sarolaner + moxidectin + pyrantel and efficacy relative to placebo (Studies 4 and 5)

Tick strain origin^a^	Count day	Placebo		1.2 mg/kg sarolaner + 24 µg moxidectin + 5 mg/kg pyrantel			Test statistic (*vs* placebo)
		Mean	Range	Mean^b^	Range	% Efficacy	
Slovakia, Germany and Ireland (Slovakia and Ireland)	2	15.4	9–20	0	0	100	*t*_(15.3)_ = 31.2 *P* < 0.0001
	7	14.3	12–18	0	0	100	*t*_(11.8)_ = 57.63 *P* < 0.0001
	14	14.0	10–16	0	0	100	*t*_(9.4)_ = 46.63 *P* < 0.0001
	21	16.1	13–20	0	0	100	*t*_(13.8)_ = 54.8 *P* < 0.0001
	28	14.9	13–20	0	0	100	*t*_(12.8)_ = 55.78 *P* < 0.0001
	35	15.4	10–20	0	0	100	*t*_(14)_ = 27.15 *P* < 0.0001
Germany (Germany)	2	15.9	3–25	0	0	100	*t*_(14.3)_ = 13.25 *P* < 0.0001
	7	14.0	9–18	0	0	100	*t*_(14.2)_ = 38.09 *P* < 0.0001
	14	16.4	10–22	0	0	100	*t*_(14.2)_ = 35.42 *P* < 0.0001
	21	15.6	10–21	0	0	100	*t*_(14.2)_ = 32.02 *P* < 0.0001
	28	17.6	11–23	0	0	100	*t*_(13.8)_ = 32.93 *P* < 0.0001
	35	18.0	15–20	0.5	0–4	97.2	*t*_(14.1)_ = 13.34 *P* < 0.0001

^a^Countries from which ticks were originally isolated to establish colony (countries of origin for ticks subsequently used to enrich colony the last time 1 and 2 years before study conduct, respectively)

^b^Mean live tick count significantly lower than placebo (Study 4: 17.16 ≤ *t*_*df*_ ≤ 63.54, 9.4 ≤ *df* ≤ 15.3; Study 5: 13.25 ≤ *t*_*df*_ ≤ 38.09, 13.8 ≤ *df* ≤ 14.3, *P* < 0.0001) at all time-points

### *Rhipicephalus sanguineus*

Placebo-treated dogs maintained adequate tick infestations throughout the study with mean tick recoveries ranging from 48–55% of the applied infestation (Table 5). Efficacy against an existing infestation was 100% and efficacy against subsequent weekly re-infestations was ≥ 97.2% through Day 35. Mean tick counts were significantly lower than placebo on all post-treatment counts (15.55 ≤ *t*_*71.8*_ ≤ 17.79, *P* < 0.0001).

**Table 5:** Arithmetic mean live *Rhipicephalus sanguineus* counts for dogs dosed once orally with sarolaner + moxidectin + pyrantel and efficacy relative to placebo (Study 6)

Count day	Placebo		1.2 mg/kg sarolaner + 24 µg moxidectin + 5 mg/kg pyrantel			Test statistic (*vs* placebo)
	Mean	Range	Mean^a^	Range	% Efficacy	
2	24.5	15–36	0	0	100	*t*_(71.8)_ = 17.79 *P* < 0.0001
7	24.3	19–32	0.3	0–2	99.0	*t*_(71.8)_ = 17.07 *P* < 0.0001
14	27.4	11–42	0.6	0–2	97.7	*t*_(71.8)_ = 15.85 *P* < 0.0001
21	25.4	15–37	0.4	0–2	98.5	*t*_(71.8)_ = 16.67 *P* < 0.0001
28	24.4	18–32	0.6	0–3	97.4	*t*_(71.8)_ = 16.09 *P* < 0.0001
35	26.4	19–35	0.8	0–2	97.2	*t*_(71.8)_ = 15.55 *P* < 0.0001

*Note*: Dogs infested with ticks from colony originated with ticks from France and subsequently enriched with ticks from France and Greece, the last time 1 year before study conduct

^a^Mean live tick count significantly lower than placebo (15.55 ≤ *t*_*71.8*_ ≤ 17.79, *P* < 0.0001) at all time-points

## Discussion

These studies demonstrate that a single oral dose of the new combination product providing a minimum of 1.2 mg/kg sarolaner, 24 µg/kg moxidectin and 5 mg/kg pyrantel is effective against the ticks commonly infesting dogs in Europe. Against existing infestations of *D. reticulatus*, *I. hexagonus*, *I. ricinus* and *R. sanguineus* (*s.l.*) the combination product reduced live tick counts by ≥ 98.9% at 48 hours after treatment. Against weekly re-infestations live *D. reticulatus* counts were reduced by ≥ 97.2% at 48 hours after infestation for 28 days, and live *I. hexagonus*, *I. ricinus* and *R. sanguineus* counts by ≥ 97.2% for 35 days.

Efficacy results for the combination product in the current studies are comparable to those for similarly designed laboratory studies that evaluated the efficacy of single active isoxazoline parasiticides against the same tick species. In published studies, against existing infestations of *D. reticulatus*, *I. ricinus* or *R. sanguineus*, a single oral dose of afoxolaner [11, 12] or lotilaner [13] provided ≥ 96.0% reduction in geometric mean tick counts relative to placebo 48 hours after treatment, and ≥ 95.1% reduction at 48 hours after weekly re-infestations for at least 28 days.

Interestingly, for the majority of ectoparasiticides, efficacy against *I. hexagonus* has not been demonstrated. The importance of *I. hexagonus* is evident from data published in prevalence studies, showing that up to 8.8–39.0% of examined dogs are infected with this species [14–17]. In the UK, it is consistently reported as the second most prevalent tick species infesting dogs after *I. ricinus* [14, 17]. Furthermore, *I. hexagonus* seems to be as frequently infected with common tick-borne pathogens such as *B. burgdorferi* (*s.l.*), *A. phagocytophilum* and *Rickettsia* spp. as *I. ricinus* [15, 18], while the transmission of these pathogens to dogs by *I. hexagonus* has not yet been documented.

The tick species investigated here are endemic throughout Europe and the geographical range and seasonality of infestation for these ticks appears to be expanding, at least in part due to changes in climate [19]. The tick-borne pathogens these tick species may transmit, can lead to clinical disease in dogs [1, 4]. Properties of the host, tick, pathogen and climate all can contribute to variability in the time between tick attachment to the host for feeding and the transmission of pathogens [20]. The period of time between tick attachment and pathogen transmission results in a window of opportunity for which removing or killing ticks will reduce or eliminate disease agent transmission [21, 22]. Removing or killing ticks within 36–48 hours of attachment should reduce the potential for the transmission of some of the tick-borne pathogens e.g. *Babesia* spp. [21].

In addition to ticks, other ecto- and endoparasites may also infect dogs. The European Scientific Counsel Companion Animal Parasites (ESCCAP) considers fleas and ticks to be prevalent ectoparasites, and the ascarids, *D. immitis* and *A. vasorum* to be ‘key’ endoparasites of dogs in Europe [1, 5]. Due to the high prevalence of these parasites in some or all of Europe, and their ability to directly or indirectly cause significant clinical disease in the dog or pose a zoonotic risk to humans, ESCCAP recommends sustained treatment of dogs at risk for these parasites [1, 4, 5].

The novel combination of sarolaner, moxidectin, and pyrantel in Simparica Trio™ provides for efficacy against these common external and internal parasites in a single oral chewable tablet. The studies presented here confirm the efficacy of sarolaner at a minimum dose of 1.2 mg/kg against the common tick species infesting dogs in Europe, and additional studies confirm its efficacy against fleas [23], the efficacy of moxidectin against *D. immitis* [24] and *A. vasorum* [25], and the efficacy of pyrantel against gastrointestinal nematodes [26, 27].

## Conclusions

A single oral administration of Simparica Trio™ providing minimum dosages of 1.2 mg/kg sarolaner, 24 µg/kg moxidectin and 5 mg/kg pyrantel pamoate was well tolerated and efficacious for at least one month against the four tick species commonly infesting dogs in Europe.

## Data Availability

Data upon which the conclusions are based are provided within the article.

## Abbreviations

ESCCAP: European Scientific Counsel Companion Animal Parasites
WAAVP: World Association for the Advancement of Veterinary Parasitology
